# Community-integrated noncommunicable disease service models: lessons from China

**DOI:** 10.7189/jogh.15.04296

**Published:** 2025-10-03

**Authors:** Hongyi Xu, Min Liu, Yamin Bai, Jing Yang, Yueru Liu, Xinlei Gao, Alarcos Cieza, Jing Wu

**Affiliations:** 1 World Health Organization, Department of Noncommunicable Diseases, Rehabilitation and Disability, Geneva, Switzerland; 2 National Center for Chronic and Noncommunicable Disease Control and Prevention, Chinese Center for Disease Control and Prevention, Beijing, China; * Joint first authorship.

## Abstract

**Background:**

Integrated health services are advocated for primary health care to address non-communicable diseases (NCDs), especially in low- and middle-income countries (LMICs). However, evidence of care delivery models and means of scaling up is limited. This study examines community-integrated NCD service models in China, providing evidence on the steps that China took to reorient health services towards primary health care to address the NCD epidemic.

**Methods:**

A systematic review identified and included 20 studies (from 3959 records screened from five databases) conducted in various regions of China, published in English or Chinese. The evidence synthesis is narrative and thematic. Themes are built upon from the Chronic Care Model framework and issues identified in the World Health Organization package of essential noncommunicable (PEN) disease interventions. They cover priority diseases, interventions included in packages of care, delivery strategies at the community level, the roles of stakeholders, approaches to overcome health system challenges, outcomes, and gaps.

**Results:**

Despite facing common challenges like other LMICs, such as inadequate infrastructure and insufficient human resources, various community-level integrated NCD service models have been trialled and scaled up through health reform and policy implementation. Key interventions include health promotion, screening, tiered diagnosis and treatment, patient education, self-management, and digital health models. Family physicians and nurses are the main providers, supported by local governments and hospitals. The review identified creative service delivery strategies at the community, highlighting changes in patient clinical pathways, improved access to services, and positive clinical outcomes.

**Conclusions:**

China's experience with community-integrated NCD service models offers valuable insights for other LMICs. Key elements include prioritising universal health coverage, integrating public health and primary care, and optimising accessibility, efficiency, and patient-centredness. Future research should focus on long-term effects and sustainability, particularly in rural settings.

Non-communicable diseases (NCDs), such as diabetes, cardiovascular disease, cancer, and chronic respiratory disease, account for 74% of all deaths worldwide [1]. To tackle this issue, especially in low-resource settings, the World Health Organization (WHO) recommends prioritising and delivering a set of cost-effective interventions through an integrated approach [2] and financing essential interventions of NCDs within the universal health coverage (UHC) agenda.

The WHO defines integrated health services as those that are managed and delivered so that people receive a continuum of health promotion, disease prevention, diagnosis, treatment, disease management, rehabilitation, and palliative care services. These services are coordinated across different levels and sites of care within and beyond the health sector, tailored to individuals’ needs throughout the life course [3]. However, implementing NCD interventions through an integrated approach in low- and middle-income countries (LMICs) with weak primary health care systems is challenging. Additionally, there is limited evidence on the implementation and delivery of such integrated strategies in these contexts [4].

The People’s Republic of China, a developing country marked by rapid urbanisation and economic growth, has lifted more than 850 million people out of poverty. Life expectancy at birth increased from 69.3 years in 1990 to 77.4 years in 2019 [5]. With one of the fastest aging populations, China had approximately 258 million people aged 60 and over in 2021 [6].

Recognising the burden of noncommunicable diseases in its aging society, which undermines its social and economic development, the country launched health reforms towards UHC in 2009 through the National Basic Public Health Services (BPHS) Programme. NCDs were gradually included and expanded, as reflected in the list of services and interventions, priority diseases, coverage of the population, and financial coverage [7–9].

In 2017, the BPHS purchasing arrangement expanded from nine to 14 categories, including packages for screening, referrals, and following up on hypertension, type 2 diabetes, severe mental disorders, tuberculosis, and care for older people. Recently, the programme further expanded to include services for chronic obstructive pulmonary disease (COPD) patients. Funding for the programme increased from 3.10 USD per capita in 2009 to 7.00 USD in 2017 and 13.00 USD in 2022 [7]. The central government covers up to 80% of the total BPHS budget for 12 low-income provinces and only 10% for two high-income areas (Beijing and Shanghai).

Like other LMICs, reorienting primary health care to deliver integrated NCD services remains challenging. Implementation is constrained by inadequate infrastructure, insufficient human resources, medicine availability and affordability, improper health information systems, poor quality, and a lack of trust in first-level services [10–13]. In China, the medical service system is largely hospital centred. While China’s health reform prioritises strengthening primary health care, it also examines the role of hospitals in this process [14].

Various integrated NCD service models at the community level have been trialled and piloted. Many localities have scaled up these pilots through the ‘demonstrative districts’ of the National Demonstration Areas Program for Integrated NCD Prevention and Control [15]. In this study, we examine elements of community-integrated NCD service models in China, focusing on prioritised diseases and their interventions, the organisation and coordination of providers, and the management of services along the care pathway. We examine the extent to which NCD services have been integrated and coordinated among levels and sites of care through a primary health care and patient-centred lens.

We hope that our analysis will provide valuable insights into integrated care and community-based health care models, offering lessons for other LMICs.

## METHODS

We followed nine defining features of conducting a systematic review, including articulating objectives, defining inclusion and exclusion criteria, conducting a comprehensive search, following a screening and selection process, appraising the quality of studies, analysing extracted data, presenting a synthesis of the results/findings, interpreting the results, and reporting transparently [16].

The synthesis is both narrative and thematic. Its structure is built upon themes of interventions and types of services provided through the NCD integrated service models identified in a previous scoping review [17]. These themes are also related to health systems, self-management, and a patient-centred disease management approach, as identified in the Chronic Care Model framework [18]. Additionally, they consider the issues identified in the WHO package of essential noncommunicable (PEN) disease interventions [2].

The WHO PEN [2] features common NCD management (such as hypertension, diabetes, asthma and COPD, and the early cancer diagnosis), lifestyle counselling, self-care, and palliative care. In our previous scoping review [17], at the community level (including health centres, typical health facility in the community), the frequent reported service types were health education, health promotion, screening, adherence support, home visits, initial diagnosis, patient follow-up, and medication dispensing.

The review protocol was registered in the PROSPERO Database (CRD42023420692). We report our implementation of the PRISMA-P (Preferred Reporting Items for Systematic Review and Meta-Analysis Protocols) guidelines (Table S1 in the **Online Supplementary Document**). We also compared the thematic synthesis of the community-integrated NCD model in China to other similar works, discussing contextual issues.

### Criteria for considering studies in this review

A predefined set of eligibility criteria was used to screen studies for inclusion in the review (Table S2 in the **Online Supplementary Document**). The research was restricted to community in China. Inclusion and exclusion criteria for the selection of studies for the review were found in Table S2 in the **Online Supplementary Document**. The study population was defined as people with chronic diseases in the community, and the integrated NCD model was defined as the delivery of chronic disease management services in the community, where patients received services that include a variety of components, including health promotion, disease screening, diagnosis, treatment and rehabilitation. These services can be coordinated between different levels of health services in response to the different needs of patients and stages of disease. Included studies were required to be scientifically designed and able to provide data to support the assessment results.

We searched the following electronic databases: Medline, Excerpta Medica database (EMBASE), the Cochrane Library, CINAHL, and the Chinese Index Medicus. The search details are available (Table S3 in the **Online Supplementary Document**). To supplement our search, we also conducted a manual search and reviewed reference lists of identified studies and reviews.

### Study screening and selection

The data were downloaded and screened in NoteExpress. Studies were first screened by reviewing titles and abstracts by two authors. Where applicable, the full texts of relevant studies identified through a second screen of studies were obtained, and the data were extracted into a piloted Excel spreadsheet. The research was restricted to communities in China.

### Data extraction and quality appraisal

The following data were extracted from the included studies: first author, year of publication, types of NCD, study design, sample size, population characteristics, location, health service setting, health service context, outcome measures, details of intervention, and summary of results. The extracted items are summarised in the results.

Two authors extracted the data. One author conducted a quality assessment for the selected studies using the Cochrane ROBINS-I tool (to assess the risk of bias in the results of nonrandomised studies) and the Joanna Briggs Institute (JBI) checklist. Data extraction forms were piloted on a sample of included studies (20%) to ensure all the relevant information was captured. The consistency of the extracted data was assessed to ensure >95% agreement.

### Data analysis and synthesis

We conducted a narrative synthesis of studies, including the type of study, location, measurements and indicators, and characteristics of providers. We map and match our findings and draw our themes deductively based on established principles and themes, such as the WHO PEN framework and the Chronic Care Model. For services provided through a community integrated model, we mapped each study against a list of types of services provided through a previous scoping review [17].

Our other syntheses are narrative, focusing on implementation aspects, including delivery strategies at the community level, the roles of stakeholders, approaches to overcome health system challenges, outcomes, and gaps. Revisions of the synthesis involved looking for relationships and common characteristics among the themes. We presented our results using summary tables, graph highlighting the change of patient clinical pathways, and narratives.

## RESULTS

**Figure 1:**
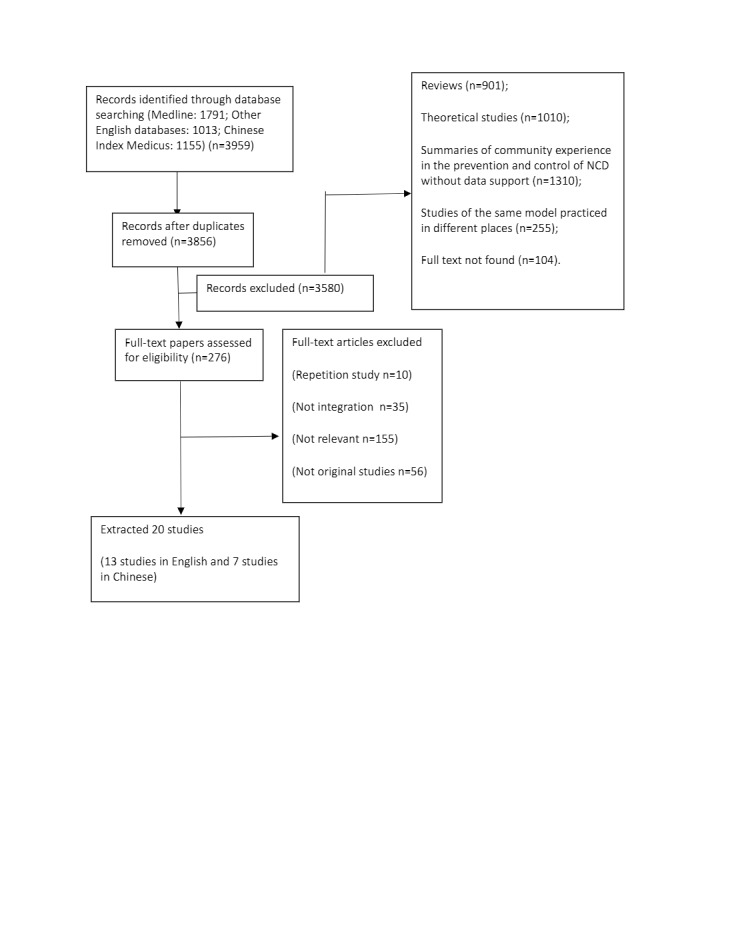
PRISMA flowchart detailing the results of the literature search and screening of primary research studies

Our initial search included 3959 papers, which were reduced to 3856 after deduplication. After screening titles and abstracts, 276 studies remained. Of these, 155 studies were found to be irrelevant; 56 studies did not use primary data, including reviews and policy briefs; 10 studies were excluded due to repetition; and 35 studies were not integration related. This left 20 studies (**Figure 1**).

Of the 20 included studies, 16 were conducted in East China, two nationwide, one in West China, and one in the middle region. Among the studies specifying service delivery location, 10 were conducted in urban areas, two in rural settings, and eight in mixed regions. Of the 20 included studies, four have sample size less or equal than 100, eight between 100 and 1000, eight more than 1000 (of which three time series studies have sample size more than 8000). Eight time-series studies present more than two years long-term follow-up data (**Table 1**). Our selected studies include a mix of experimental studies (8 studies) and quasi-experimental or observational studies, including assessments of the effect of China’s BPHS policy implementation and a national demonstration of the integrated NCD prevention and control programme (**Table 1**).

**Table 1:** Summary of the literature on community-integrated NCD prevention and control in China

No.	First author year	Study design	Sample size	Follow-up time	NCD Types	Details of model: workforce, interventions, delivery strategy	Results measurement and impact	Place
1	Li Sixuan 2023 [19]	Cohort study	502	4 y	Hypertension	Community family doctors, follow-up visits, measure blood pressure, heart rate and weight, lifestyles intervention, medication, and health education to patients.	2015 to 2019. Effect of BPHS policy. Measured by clinical outcome: blood pressure.	Ningbo (Zhejiang Province)
2	Jiaoling Huang 2019 [20]	Before-after study	1734	3 y	Diabetes, hypertension	Family doctor-contracted services, primary health centre, regular monitoring, health profile updates, treatment, health education, healthy behaviour promotion, and supporting self-management through focus groups.	2013 to 2016. Evaluation of family doctor service. Health knowledge, engagement in health behaviour, and self-monitoring. Marriage, education, hukou, medical costs, and satisfaction with PHC were significant predictors of engagement in self-management initially.	Shanghai
3	Fu Dongbo 2003 [21]	Randomised controlled trial	954	8 mo	Chronic lung disease, diabetes, hypertension, heart disease, stroke, arthritis	Effectiveness of Shanghai chronic diseases programme. Community-based patient self-management education course. Community health providers and lay-leaders. Topics include exercise, cognitive symptom management, nutrition, fatigue and sleep management, community resources, medications, dealing with emotions and depression, communication, action planning, problem-solving, and decision-making.	Health behaviours, health status, self-efficacy, self-management skill, health service utilisation, and saving in health care	Shanghai
4	Wei Kan 2021 [22]	Randomised controlled trial	96	6 mo	Diabetes	Participatory approach: community health promotion and intervention, focusing on self-management (22 items), patient education, patient follow-up, professional guidance, family supervision, and communication and support from patients peers. Funding and incentives (including funding to refurbish clinics, additional equipment and subsides for public health work).	Effect on clinical outcomes (fasting blood glucose, 2hPG and HbAlc), plus knowledge, self-efficacy, self-management level, and quality of life.	Xinjiang
5	Yong Liu 2014 [23]	Quasiexperimental, time series	8074	2 y	Chronic disease	Model adapted to less-developed rural areas in 7 townships. Engage county government and capacity building of primary health centre practitioners (health manager, doctor, nurse): training, clinic improvements, clinical guidelines, and standards. Improve management process in PHC, improve access to quality PHC, and subsidies for public health service (immunisation, home visits, patient education). Funding for equipment and repairing, accrediting.	Public health service utilization (immunisation, gynaecological examination, and antenatal examination rate). Satisfaction on patient centred care (explanation, responsiveness, and trust)	Chongyi and Luxi in Jiangxi Province, underdeveloped area
6	Yumin Zhou 2010 [24]	Cluster randomised controlled trial	1062	4 y 8 mo	COPD	Health education (smoking cessation, knowledge of COPD and risk factors), air quality, individualised intervention and patient education (smoking cessation, improve air pollution and working environment, exercise, and improve living environment such as recommendations for better stoves, kitchen ventilation, regular telephone or home visits), treatment (compound bronchodilator), and pulmonary rehabilitation, medicine use and diet, and quarterly visit, self-management.	FEV1, air pollutants, smoking cessation rate and reduced exposure to environmental tobacco smoke, knowledge of COPD and smoking hazards, outdoor air quality, environmental tobacco smoking, and working conditions.	Guangzhou (Guangdong Province)
7	J Woo 2009 [25]	Before-after study	33	5 mo	COPD and chronic heart failure	Designed by a team of doctors, nurses, and physiotherapists. Programme can be nurse-led, or led by trained supervised volunteers. Exercise prescriptions, patient education, and rehabilitation group support.	Compliance and satisfaction, knowledge, symptom, exercise tolerance.	Hong Kong
8	Jing Xu 2016 [26]	Cross-sectional comparative study	1254	One year	Diabetes type 2 and hypertension	Collaboration models between hospitals and community health centres: loose, consortium, direct management. 2 models, hospitals provide or share equipment and other resources, and managerial support to the affiliated community health centres.	Indicators of the process of collaboration models: outreach specialist, workload, training, two-way referral, stakeholders’ perception, patients’ satisfaction.	Wuhan (Hubei Province); Zhenjiang; Nanjing (Jiangsu Province)
9	Jing-Xia Kong 2019 [27]	Group randomised experimental study	258	9 mo	Diabetes type 2	Chronic care model to enhance NCD management: self-management support helping with goals setting and monthly plan, follow-up, patient education and communication, plus health information. Decision support through clinical guidelines and continuous medical education. Team-based (physician, health manager, public health assistant)	Healthy behaviour, clinical outcome (body mass index, waist circumference, fasting blood glucose, HbA1c, blood pressure, and serum lipid), and quality of life (physical and mental health component)	Hangzhou (Zhejiang Province)
10	H. Hu 2010 [28]	Cross-Sectional Comparative Study	8231	7 y	Chronic diseases (top 10)	Evaluate community health service network model for the accessibility and optimising the primary care institutions in underdeveloped parts of China, NCD services focusing on screening, health education, diagnosis. Operation emphasises infrastructure (housing, provision of medicines and equipment), personnel provision (general practitioner) and training, including in the medical insurance system, evaluation). Context: 15% NCD patients have no access to formal NCD treatment. Among access, 70% use primary health facilities. Top 10 prevalent chronic diseases (hypertension, heart disease, gastritis, cerebrovascular disease, bronchitis, degenerative disc disease, diabetes, rheumatoid arthritis, digestion and ulceration, gallstone illness)	Access to service, hypertension management (medication compliance, self-monitoring, control rate), knowledge, health behaviours.	Puyang (Henan Province), underdeveloped area
11	Li-Juan Liu 2013 [29]	Interviews and focus group discussions	960	6 mo	Diabetes	Chronic care model including self-management behaviours, glycemic control, health information and registration system, patient assessment of chronic illness care in community health centres.	Association of care model, self-management, and glycemic control. Complex disease, age, gender, and education factors on utilisation of community health centre (44%) or hospital (56%).	Shanghai
12	Yanbing Zeng 2019 [30]	Cross-sectional survey	500	1 y	Chronic diseases	Team care chronic disease management model innovatively involved specialist, family physician, and community nurse. Financial incentives (lower medicine prices and high reimbursement rates), policy of abolishment of the general outpatient services in hospitals for hypertension or diabetes, specialist making rounds, and essential medicine availability to encourage care delivered at community health centre.	Cost of treatment, visits and seeking health care in CHCs, and clinical outcome (blood pressure and glucose). Waiting time at hospital. Awareness, physician, and community members preference.	Xiamen (Fujian Province)
13	Xuejuan Wei 2018 [31]	Retrospective time series	2451	2 y 3 mo	Diabetes type 2	Intelligent chronic disease management model, elderly home care. Include artificial intelligence, patient information sharing electronically and real-time warning, clinic decision and bidirectional referral, the Internet of Things, text messaging, and internet information to inform patients and family members of risk factors and to aid self-managing and evaluation of family doctors.	Clinical outcome (total cholesterol, low density lipoprotein-cholesterol and low density lipoprotein-cholesterol, HbA1C, fasting blood glucose). Patients follow-up and test rate.	Beijing
14	Yuexing Liu 2020 [32]	Before-after studies	1284	One year	Diabetes	Integrate primary and specialty care, community and clinic based peer support, monthly patient education (diabetes) classes and activities encourage healthy lifestyles and diabetes management, led by community health centre or coled with peer leaders, WeChat peer group, Individual follow up with extra support, peer leaders interactions with families, collaborate resources in neighborhoods. Responsibility of key stakeholders, for instance, community to support peer leaders.	Clinical outcome HbA1c, diabetes distress, increased neighborhood support.	Shanghai
15	Wei Wang 2018 [33]	Randomised controlled trial	118	One year	Hypertension	Comprehensive management: establish electronic records and patient information, standardised management, a self-management group, health education, and dietary and medication guidance.	Body mass index, hypertension knowledge, blood pressure control and complications rate	Beijing
16	Han Zhang 2019 [34]	Before-after study	2400 in 2013, 2645 in 2017	4 y	Diabetes and hypertension	National demonstration for integrated NCD prevention and control. Comprehensive NCD interventions include government policy and funding, patient record and follow-up, health education and promotion (establishing healthy places, sites for self-testing of blood pressure and sugar level, healthy trails, fitness facilities, and cycle paths), and health information platform	Self-reported rate, prevalence, control rate of hypertension and diabetes	Wuhan (Hubei Province)
17	Xian Zhang 2019 [35]	Randomised controlled trial	320	10 mo	Diabetes type 2	Patients followed up regularly by the contracted general practitioners and given lifestyle suggestions (telephone sessions and/or face-to-face sessions).	Clinical outcome: blood glucose and lipids level	Shanghai
18	Cuiling Huang 2019 [36]	In-depth interviews	24	2 y 4 mo	Chronic diseases	1 + 1 + 1 (one community health centre, one district medical institution, one municipal hospital) signing model to improve two-way referral and management of chronic. Preferential policies (long-term prescription, extended prescription, hospital booking of higher-level hospitals, and referral)	Signing rate, policy implementation, medicine availability, family doctors workload, and income.	Shanghai
19	Colette Browning 2012 [37]	Before-after studies	100	12 mo	Diabetes	Community physicians and nurses conduct motivational interviewing with Type 2 diabetic clients: a guided, client-centred counselling approach to motivate behavioural change in diet and physical exercise.	Blood pressure	Beijing
20	Fei Wu 2015 [38]	Time series, programme reported data	1.5 million and 0.39 million	4 y	Diabetes and hypertension	Programme in 8 provinces. Train health workers in rural areas on disease management, tailored health education and promotion (for example, house-family-community model, school health-family-community model, demonstration family and community, health corridor), interventions to high-risk populations and patients, monitoring, and evaluation. Policy to increase reimbursement rate of treatment.	Reported management rate and control rate of hypertension and diabetes	Henan, Chongqing, Jiangsu, Gansu, Shannxi, Qinghai, Heilongjiang, Shanxi Provinces.

BPHS – National Basic Public Health Services, NCD – Noncommunicable disease, COPD – Chronic Obstructive Pulmonary Disease, CHC – Community Health Centre

### Prioritised diseases

Among the selected studies, seven targeted diabetes, six involved all types of chronic diseases, three targeted hypertension and diabetes, two targeted hypertension only, and a final two targeted COPD (including one targeting COPD and chronic heart diseases). Although multimorbidity and chronic heart diseases were included in some studies, most studies excluded patients with complications, acute phases, cancer, and terminal illness.

Hypertension and diabetes were selected as entry points for primary care and disease management in several studies [34]. Communities in Guangzhou and Hong Kong have piloted interventions for COPD and chronic heart diseases [24,25].

### Interventions and types of services

The service types identified across the studies included health promotion, health education, screening, referral, initial diagnosis, adherence support, peer-group facilitation, acute care, home-based care, home-based visits, medication distribution, patient follow-up, traditional Chinese medicine treatment, monitoring, and medication management. Health education and health promotion were reported in all the studies.

We grouped the services into five areas:

1. health promotion, health education, and developing a healthy environment;

2. screening, assessing health, and establishing records;

3. tiered disease diagnosis and treatment, patient management, follow-up, and referral;

4. patient education, lifestyle counselling, self-management, and peer support;

5. digital intelligence models including telemedicine, digital health, the internet and the Internet of Things.

Two studies included rehabilitation support in models of COPD and chronic heart failure. In practice, traditional medicine was found to be a common service in the community model [34]. HPV vaccination was provided through an existing immunisation or gynaecological programme [23] or through school.

#### Health promotion, health education, and developing a healthy environment

This area includes health literature and knowledge dissemination through community health centres and other channels targeting the whole population. Packages include diet, such as nutrition and salt reduction; knowledge of common chronic diseases, such as hypertension, diabetes, and COPD, and their risk factors; establishing exercise and a healthy environment; and health promotion at school and other entities, including households, workplaces, and restaurants [24, 34, 38].

The demonstrative districts for NCD prevention and control in China [34,38] incorporated the NCD agenda into local government plans, formed leadership groups, and provided budget lines and financial support for activities. For example, they established various ‘healthy places,’ such as sites for self-testing of blood pressure and sugar levels, walking trails, fitness facilities, cycle paths, and 15-minute fitness circle.

A study in Guangdong included interventions for indoor air pollution due to smoke (tobacco or cooking) and ventilation and outdoor air pollution. It included pulmonary rehabilitation exercise led by a community nurse, in addition to the treatment of COPD [24].

#### Screening, assessing health, and establishing health records

Screening is used as an approach for the early diagnosis of NCDs and entry into the community integrated care model to identify, plan, and stratify patients for differentiated management. Blood pressure and sugar levels are recommended to be checked when patients visit a health centre for the first time. Some community health centres provide equipment or tools for self-checking. Hypertension and diabetes patients are requested to have a cohort record established and maintained at health centres [33, 34, 38]. Periodic home visits and health assessments, including the measurement and recording of blood pressure and blood glucose levels for hypertension and diabetes patients, are needed, according to the National Basic Public Health Services Programme [7, 34].

#### Tiered disease diagnosis and treatment, patient management, follow-ups, referrals

This area forms a key effort of the government’s health reform, aiming to build a primary health care system with gatekeeping functions, decentralise resources, and shift the management of common NCDs to the community. The review revealed that diabetes, hypertension, and COPD were the common types of diseases currently managed in the community, with diabetes and hypertension serving as entry points [24, 27, 30, 31, 33, 34, 36, 38]. Various models of hospital - community health centre collaboration and team care approaches in China have been piloted to facilitate the management of common NCDs at the community level [26]. The results were heterogeneous, which will be elaborated on later in the discussion section.

#### Patient education, lifestyle counselling, self-management, and peer support

Patient education [24, 25, 27, 29, 32] evolves providing patients with information to understand the disease condition and diagnostics, support and guidance to manage their health conditions and make informed decisions, adherence to treatment, and when to call for help. Counselling for healthy lifestyles involves guiding and supporting patients in making changes in certain behaviours to reduce the risk of NCDs [29, 32-38].

Several studies in this review explored managing patients and risk factors for NCDs through signing contracts [34] between the family doctor and patients, enhancing patient education and adherence through follow-up and/or home visits. One effective approach involves forming peer-support patient groups, providing training to patients on self-management and medication compliance, and helping patients set goals and make self-management plans. This was reflected in Shanghai, where there was even a ‘diabetes club’ [21,29]. Activities and interventions, including routine exercise and/or rehabilitation exercise [24,25] and social activities, were led by community-based health workers (such as nurses) or peer leaders.

#### Intelligence models, including telemedicine, digital health, the internet and the Internet of Things

The next generation of the community health care model in China is positioned as an intelligence model that incorporates information technology, telemedicine, digital health, the internet, and the Internet of Things [31]. In some studies, an electronic system for the exchange of health information was utilised to support the delivery of integrated care and enhance the effectiveness of service delivery [8,27,31,33].

### Organisation and coordination of providers

The review revealed that family physicians (general practitioners/family doctors) and nurses in community health centres constitute the main category of the health workforce that provides the bulk of services [19–21,23,25,27,30,31,35]. In the selected nonexperimental studies, such as the studies in Shanghai [36] and Xiamen [30], the family physician or physician team was contracted to cover an administrative catchment population.

In addition to health centres as the main service provider, studies have identified two other stakeholders: local governments [23,24,26,34,38] and hospitals [26,30,36]. The local government owns and finances community health centres and supervises the operation and provision of public health services [26]. Some local governments have adopted policies and incentives to unlock hospitals’ capacity and resources to strengthen primary health care conducted by community health centres. Community and local governments provide input and support to the infrastructure and equipment required by community health centres. They also organise activities, such as health promotion among households and women, and support health education and self-management interventions led by patients’ peer support groups or non-health professional leaders [21,25,32].

Hospitals that previously provided the bulk of the diagnosis and treatment directly to chronic patients, such as those with hypertension and diabetes previously, were requested to reallocate resources and implement a tiered medical care system. The spectrum of hospital roles shifted to service outreach at community health centres, championing integrated care models [30,32], mentoring community health centres to perform medical service tasks such as the diagnosis and treatment of common NCDs, and implementing reciprocal referrals and triage.

In some pilots on reciprocal referrals, when needed and referred by community health centres, patients with complications receive fast-track services at hospitals, and patients with stable diabetes and hypertension conditions are referred to community health centres for medication refills and follow-up [26,30,36]. Physiotherapists were involved in designing intervention programmes for COPD and chronic heart failure, as reflected in the Hong Kong study [25].

At the administrative level, the three types of stakeholders often form a consortium or collaborative body, through agreements or contracts [30,36]. Studies have also identified community health service networks and joint rehabilitation centres among communities as approaches to enhance service provision and quality in economically less developed areas [23].

### Management of services along the pathway of care and different levels of care

**Figure 2:**
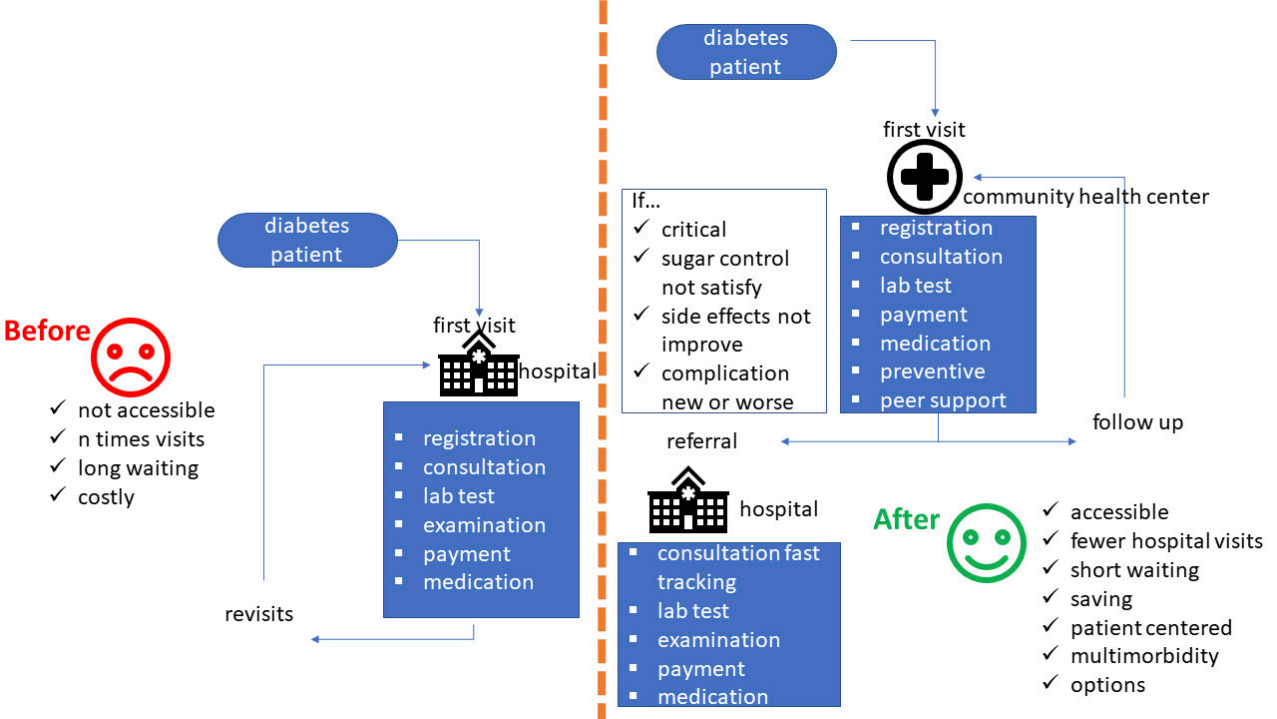
An illustration of a diabetes patient clinical pathway in Shanghai: before and after the implementation of community-integrated NCD service models

Several studies in this review examined the efforts of health care reforms in pilot cities, such as Shanghai and Xiamen, which focused on triaging patients from hospitals to community health centres and promoting the utilisation of community services. We summarise the changes in patient clinical pathways, services provided at the community and hospital levels, and the initial impact in **Figure 2**, using a diabetes patient in Shanghai [36] as an example.

As a result, several studies have identified encouraging changes in the health care-seeking behaviour of stable diabetes and hypertension patients and their preferences in pilot cities [30,31,36]. Patients now request and receive medication and routine follow-ups in community health centres due to easier access and cost savings [26,36].

### Measurement and patient-centredness

Among the 20 selected studies, 14 included clinical outcomes such as blood pressure, fasting blood glucose, HbA1c, and forced expiratory volume (FEV1) as measurements. Programme indicators, including service utilisation and preferences across the level of health care (community or hospital), referrals and patient visits, cost savings and waiting times, patient contracting rates with family doctors, and disease (hypertension and diabetes) standard management rates and control rates, were adopted as part of performance management and as accountability mechanisms. Notably, there was an interest in measuring the empowerment of patients and how patient-centred the model was. Various indicators, including composite indicators such as stress and neighbourhood support, self-efficacy, self-management, patient satisfaction, and quality of life, were adopted to present evidence of the impact of an integrated model (**Table 1**, **Table 2**).

**Table 2:** Summary of the outcome measurement on community-integrated NCD prevention and control in China

Outcome or subgroup title	Measurement	No. of studies	No. of long-term studies (1 y or more)
1.1 Improvement in NCD control	Measured by fasting blood glucose, HbA1c, blood pressure, serum lipid, cholesterol, FEV1	14	10 (5 of them are ≥4 y time series)
1.2 Access to service	Measured by PHC service utilisation, consultation, patient filing, health examination, workload	6	5 (2 of them are ≥4 y time series)
1.3 Efficiency of health system	Measured by hospitalisation, complication reduction	3	2*
1.4 Patient satisfaction	Measured by patient view of service, satisfaction, awareness of reform or health care policy, waiting time at hospital, satisfaction of referral, satisfaction of health care environment, clarity of explanation of illness, responsiveness and trust	6	5 (1 of them are ≥4 y time series)
1.5 Improvement in self-management, self-efficacy	Measured by improved knowledge of disease and risk factors, blood pressure or blood glucose monitoring, medication and medication adherence	9	5 (2 of them are ≥4 y time series)
1.6 Improvement in health status, quality of life	Measured by improved health, symptom improvement, quality of life (physical and mental health component)	6	2 (2 of them are ≥4 y time series)
1.7 Improvement in healthy behaviour	Measured by physical activity, drinking, smoking cession, diet, BMI, waist circumference, household or occupational exposure to dust/gases/fumes.	5	2 (1 of them are ≥4 y time series)
1.8 Cost	Measured by cost of treatment, cost benefit or saving	2	1

*Increased hospitalisation in one study in less-developed area

Patient satisfaction, access to service, cost benefit, and efficiency in terms of reduction of hospital admissions for diabetes and hypertension, and reduction of complication were adopted as important outcomes for the models of integrated care in China [26,30,36]. The initial phase of the China community integrated model received various successes measured by those outcomes, some of them are measured by several studies after long-term follow-ups (**Table 2**). We also discuss this in the next section.

## DISCUSSION

The studies in this review provided evidence on the steps that China took in its journey to reorient health services towards primary health care to address the NCD epidemic. We discuss the lessons learned from the experience of China thematically, covering priority diseases, interventions included in packages of care, delivery strategies at the community level, the roles of stakeholders, approaches to overcome health system challenges, outcomes, and gaps and limitations. We compare the experiences of other countries with similar or different contexts.

### Priority diseases, package of interventions

With increasing coverage of NCD services in primary care, China has focused mainly on hypertension and diabetes patients, who had been inefficiently treated in hospitals until then. Once those services provide desirable outcomes, additional services, such as cervical cancer and breast cancer screening and COPD, are expanded to cover them. This approach of progressively increasing and delivering heterogeneous interventions and services at primary health centres has been found in many settings, not only in relation to NCDs but also in other areas, such as reproductive, maternal and child health programmes [17,39,40].

When defining the spectrum of the service package at the primary health care level, this review identified a mix of public health and primary medical care interventions. This represents a change compared to the past, when only public health services for infectious diseases were provided at this level in China. Screening and health assessment were used as initial public health approaches to assess the catchment population and stratify specific populations for the early diagnosis of NCDs and follow-up for a continuum of care. Other countries, such as Brazil, have a similar mix of public and primary medical care interventions in their primary health care reform, with a similar approach to delivering services [41]. However, studies in other countries also indicate that to determine an effective screening strategy, resources, including disease prevalence and screening technology, are important parameters to consider [42,43].

Another approach utilised in China to reduce the high undiagnosed rate for diabetes and hypertension is to provide free blood pressure and glucose checking for the population in community health centres in some localities. Additionally, free health assessment is provided annually to 65 years and older. These practices align WHO member states in a better position to achieve targets by 2030: 80% of people with diabetes are diagnosed; 80% of people with diagnosed diabetes have good control of glycaemia; 100% of people with type 1 diabetes have access to affordable insulin and blood glucose self-monitoring.

### Delivery strategy

To deliver services more effectively, this review identified patient peer group support as a good delivery strategy to enhance lifestyle counselling and patient management [21,22,27,29,32]. This delivery strategy not only improved clinical outcomes (such as HbA1c reduction for diabetes patients and a reduction in hospital stays) but also empowered patients by enhancing their knowledge, enabling them to manage diseases and disease distress themselves, and improving neighbourhood support and engagement through group classes, aerobic exercise, and social activities.

Countries implementing the WHO PEN for NCDs reported challenges related to lifestyle habits and adherence to medicine, including a lack of cooperation by patients and a lack of resources, training, and motivation and time among health workers to implement interventions [44]. Delivery strategies through peer support in China provide valuable experiences for other countries and communities to address these challenges. Good practices include defining and selecting peer leaders, curriculum training, group goal-setting and facilitation skills, monthly activity implementation, and policies for expansion through clinical and community settings [32].

Similarly, an evidence synthesis in the UK suggested that those ‘lifestyle advisors’, involved in paid or voluntary work with an individual or group of peers, were cost-effective in chronic care and smoking cessation [45].

### The role of hospitals in the pathway of care and primary health care system reform

After defining a service package, China focused on strengthening the health system and structure through reform and investment. This review highlights the important role of hospitals and how their resources are harnessed to improve the provision and quality of primary health care.

Practices included abolishing general outpatient services for patients with hypertension or diabetes and incentivising patients to receive medicines at community health centres; redesigning district hospitals as regional health centres; promoting close cooperation and communication between hospitals and community health centres; and increasing awareness among specialists, community physicians, community members, and patients about those reforms [26,30,36].

Indeed, a review in the Western Pacific region supported the role of hospitals in strengthening primary health care, also calling for further empirical inquiry into the impact of incentives for engaging hospitals in improving primary health care [13]. Given that hospital dominance in care delivery in LMICs and entrenched interests, health care reform, medical insurance reform, medicine reform, government investment and regulations must prioritise primary health care, community medical care, hospital-community cooperative medical care.

### Health system inputs: health information

At the operational level, the review revealed that health information and health workforce were key health system inputs for implementing community-integrated noncommunicable disease service models. Studies in the review [27,29,33,34] highlighted that effectively exchanging health information across the levels of care was critical to reach, divert, and follow up with the targeted population. Other review studies also revealed that the effective use of health information systems and digital tools facilitates the successful implementation of multiple screening programmes and care models for multimorbidity in an aging population [46,47].

China moves fast in digital technology. Patients are benefiting from the integration of digital technology and health care services, the era of digital health. Procedures such as appointments and follow-ups, consultations are conducted manually and digitally, which has enhanced access and efficiency. However, certain groups, such as elderly, less-educated, people live in deprived area with less internet connection or poor areas, also face ‘digital divide’ [48]. They rely help from younger generation or others to use digital technology. They enjoy less the benefit of digital health, further exacerbate health inequality.

Since 2014, the Chinese government has introduced policies to guide the development of digital health and its utilisation. Other approaches to address inequalities include infrastructure investment, the new service development considering digital exclusion, peer support or intergenerational mentoring or localised digital champion addressing essential digital skills among vulnerable groups, incentivise partnership and multisectoral cooperation [48–52].

### Health system input: Health workforce and infrastructure

The paper revealed that family physicians (general practitioners/family doctors) and nurses are the main PHC workforces. Like other LMICs, China also faces shortages of trained health care providers, and uneven distribution. Workforce capacity and infrastructure are better distributed in cities with better economic development and in the eastern region [53]. To address these challenges, this review (**Table 1**) and other studies [53–55] pointed out the importance of policy levers and collaborations, to allocate resources, train and accredit and fund PHC facilities in more deprived areas. Other recommendations include infrastructure investments (health facility and working area, provision and repairing of equipment, provision medicine), improving household living environment such as recommendations for better stoves, and kitchen ventilation (**Table 1**).

Notably, this review identified some creativeness, such as enlisting non-health professional lay leaders to support self-management and healthy lifestyle among patient groups [56] and various compositions of team-based care. The multicadre, multidisciplinary health workforce suggested the minimum number of roles to play:

– general practitioner (family doctor): diagnosis, prescription of medicines and tests, medication titration;

– nurses: follow-up and patient management; health assessment and record establishment; implementation of some rehabilitation exercise, possibly with lay leaders;

– public health workers: health promotion and preventive activities;

– pharmacist: medication management for NCD patients with multimorbidity;

– physiotherapist: design of rehabilitation programmes for patients.

These findings largely align with the WHO recommendations for team-based care [57]. Additionally, our review emphasised that specialists in hospitals should play roles in integrated care and strengthening medical services at community health centres [26,30,36]. The roles include:

– champion integrated care delivery;

– mentor mid-level professionals at community health centres;

– receiving referrals and providing medical care for highly complex patients;

– triaging patients to divert stable patients to community health centres;

– providing outreach services to patients at community health centres.

Other reviews have argued that NCD care is too frequently provided by a specialist focused on treatment, and that there is an urgent need to scale up public sector reform to ensure a sufficient number health workforce cadres and adequate distribution at the primary health care level [14,58].

### Outcomes and gaps

Our results revealed promising examples of essential NCD interventions targeting both the population and individuals, effectively implemented at primary health facilities. Positive outcomes include increasing access to NCD services, control of NCDs, and reducing the prevalence of risk factors, such as smoking and alcohol consumption, although more longer-term effects and sustainability are yet to be seen.

Programme data indicate a positive trend since the implementation of reform and the National Basic Public Health Services Programme, with approximately 70% of diabetes patients receiving standardised management through the programme [7,59]. At the population level, the standardised rates for awareness, treatment, and control of hypertension among men and women aged 18–69 years in China increased from 31.6, 25.5, and 6.7%, respectively, in 2007 to 38.3, 34.6, and 12.0%, respectively, in 2018 [60]. Compared with the control non-demonstrative area, the NCD programme and its multisectoral collaboration have impacted reductions in smoking prevalence, alcohol consumption, and passive smoking, with effect lasting 6–7 years [15].

Continuing the journey to address the NCD epidemic through primary health care is essential to tackle the remaining challenges and gaps. For example, the 2018 national population-based survey revealed that only 11.0% of patients with hypertension had controlled blood pressure, 31.5% of patients with diabetes had controlled blood glucose levels at any level of care, with even poorer results reported for patients in rural areas.

Gaps in access to care, quality challenges, and constraints in primary health care in rural areas remain severe [7,61]. The allocation of health resources not only affected health of residents, but also plays an important role in the sustainable development of medical and health services. A study in China showed that resources for NCD prevention and control are better distributed in cities with better economic development and in the eastern region, and that there are regional and urban-rural imbalances [53]. In order to promote equity, a series of policies were released by the government. Policies and the implementation, including increased investment in primary health care, the issuance of standardised community guidelines for the management and control of chronic diseases, the expansion of the capacity to educate and train health care providers, and the increase of incentives for individual practitioners, will bring long-run impact [54,55].

Disparities in access to health services due to social determinants are one of the major remaining challenges globally. Previous research has shown that socioeconomic status is the main determinant of chronic disease prevention and control in populations [62]. Studies in our paper [20,29] indicate that age, gender, and education are important factors of using PHC service, engaging self-management [20]. Investing high quality and equitable primary health care, subsidy and ensuring availability of affordable essential medicines are examples of effective ways to reduce NCDs burdens and inequalities [22,23,63].

### Limitations of the study

This review noted a lack of studies focused specifically on rural settings [23], limiting our ability to generate care models for rural areas or more resource-poor setting in LMICs. Future research, particularly implementation research, is required to establish the success and feasibility of community-integrated NCDs models in such setting and health systems in LMICs.

Nevertheless, we summarise various findings of included studies in rural setting to response NCDs: training health workers, policy to improve access to services, allocate resources for infrastructure and equipment, and additional financing. For instance, one earlier study [38] emphasised training health workers in rural areas on disease management and health education and promotion. The project also supported policies to improve overall access to health care and additional financing. A study on improving community health service models in less developed rural areas revealed an increase in the use of public health services at community health centres [23] but also an increase in hospital use (the ‘hospitalisation rate’), which is different compared to the urban setting. The change may be related to a lower initial hospitalisation rate, as rural population had more constraints to access before the study [28].

Our literature screen and selection may have missed literatures. We also noticed that some good practices in provinces are not formally published. Although time series data from national NCD surveillance and population surveys are used in some evaluation studies, some outcomes are programme data or reported data with quality issues. Overall, studies were conducted on a small to medium scale. The long-term contribution and effects of these actions are yet to be investigated, indicating future research directions.

## CONCLUSIONS

This review conducted a thematic analysis of the community integrated model for NCDs in China.

At the policy level, it emphasises universal health coverage through provision of prevention, health promotion, and essential NCD disease management interventions and enhancing access at primary health care level. In practice, significant efforts were made to integrate public health and primary care into the service package delivered at primary health care level. The review characterises how hospitals, community health centres, the community, and local government collaborate to provide health care services and perform public health functions at primary health care level in China. This review identifies the key elements of the model, which largely align with the WHO PEN and an integrated approach tailored to its own context. Optimising accessibility, efficiency, and patient-centredness were positioned as primary objectives in the initial phase of the implementation.

## Additional material


Online Supplementary Document
