# Supplementary material for: Community-integrated noncommunicable disease service models: lessons from China
Source: J Glob Health. 2025 Oct 3;15:04296. doi: 10.7189/jogh.15.04296 (PMC12491905; doi:10.7189/jogh.15.04296)
Supplement: Online Supplementary Document [file jogh-15-04296-s001.pdf]

Supplement to: Xu H, Liu M, Bai Y, Yang J, Liu Y, Gao X, Cieza A, Wu J. Community-integrated noncommunicable disease service models: lessons from China. J Glob Health. 2025;15:04296.

PRISMA 2020 Checklist

Section and Topic	Item #	Checklist item	Location where item is reported
**TITLE**			
Title	1	Identify the report as a systematic review.	Methods
**ABSTRACT**			
Abstract	2	See the PRISMA 2020 for Abstracts checklist.	Abstract
**INTRODUCTION**			
Rationale	3	Describe the rationale for the review in the context of existing knowledge.	Background
Objectives	4	Provide an explicit statement of the objective(s) or question(s) the review addresses.	Page (pg)3, paragraph(prg) 6
**METHODS**			
Eligibility criteria	5	Specify the inclusion and exclusion criteria for the review and how studies were grouped for the syntheses.	Methods, additional file 2
Information sources	6	Specify all databases, registers, websites, organisations, reference lists and other sources searched or consulted to identify studies. Specify the date when each source was last searched or consulted.	Methods, Search methods, pg4
Search strategy	7	Present the full search strategies for all databases, registers and websites, including any filters and limits used.	Searching strategy, additional file 3
Selection process	8	Specify the methods used to decide whether a study met the inclusion criteria of the review, including how many reviewers screened each record and each report retrieved, whether they worked independently, and if applicable, details of automation tools used in the process.	Methods, study screening and selection, pg 4-5
Data collection process	9	Specify the methods used to collect data from reports, including how many reviewers collected data from each report, whether they worked independently, any processes for obtaining or confirming data from study investigators, and if applicable, details of automation tools used in the process.	Methods, data extraction and quality appraisal, pg 5
Data items	10a	List and define all outcomes for which data were sought. Specify whether all results that were compatible with each outcome domain in each study were sought (e.g. for all measures, time points, analyses), and if not, the methods used to decide which results to collect.	Methods, data extraction and quality appraisal, pg5, prg2
	10b	List and define all other variables for which data were sought (e.g. participant and intervention characteristics, funding sources). Describe any assumptions made about any missing or unclear information.	Methods, data extraction and quality appraisal, pg5, prg2
Study risk of bias assessment	11	Specify the methods used to assess risk of bias in the included studies, including details of the tool(s) used, how many reviewers assessed each study and whether they worked independently, and if applicable, details of automation tools used in the process.	Methods, data extraction and quality appraisal, pg5, prg3
Effect measures	12	Specify for each outcome the effect measure(s) (e.g. risk ratio, mean difference) used in the synthesis or presentation of results.	N/A
Synthesis methods	13a	Describe the processes used to decide which studies were eligible for each synthesis (e.g. tabulating the study intervention characteristics and comparing against the planned groups for each synthesis (item #5)).	Method, p4 prg2-3; Method, data synthesis p5
	13b	Describe any methods required to prepare the data for presentation or synthesis, such as handling of missing summary statistics, or data conversions.	N/A
	13c	Describe any methods used to tabulate or visually display results of individual studies and syntheses.	Method, data

Section and Topic	Item #	Checklist item	Location where item is reported
			synthesis pg5
	13d	Describe any methods used to synthesize results and provide a rationale for the choice(s). If meta-analysis was performed, describe the model(s), method(s) to identify the presence and extent of statistical heterogeneity, and software package(s) used.	Method, data synthesis pg5
	13e	Describe any methods used to explore possible causes of heterogeneity among study results (e.g. subgroup analysis, meta-regression).	N/A
	13f	Describe any sensitivity analyses conducted to assess robustness of the synthesized results.	N/A
Reporting bias assessment	14	Describe any methods used to assess risk of bias due to missing results in a synthesis (arising from reporting biases).	N/A
Certainty assessment	15	Describe any methods used to assess certainty (or confidence) in the body of evidence for an outcome.	Methods, data extraction and quality appraisal, pg5
**RESULTS**			
Study selection	16a	Describe the results of the search and selection process, from the number of records identified in the search to the number of studies included in the review, ideally using a flow diagram.	Methods, study screening and selection; Figure 1, PRISMA flow diagram
	16b	Cite studies that might appear to meet the inclusion criteria, but which were excluded, and explain why they were excluded.	Not provided
Study characteristics	17	Cite each included study and present its characteristics.	Table 1
Risk of bias in studies	18	Present assessments of risk of bias for each included study.	Not provided
Results of individual studies	19	For all outcomes, present, for each study: (a) summary statistics for each group (where appropriate) and (b) an effect estimate and its precision (e.g. confidence/credible interval), ideally using structured tables or plots.	N/A
Results of syntheses	20a	For each synthesis, briefly summarise the characteristics and risk of bias among contributing studies.	N/A
	20b	Present results of all statistical syntheses conducted. If meta-analysis was done, present for each the summary estimate and its precision (e.g. confidence/credible interval) and measures of statistical heterogeneity. If comparing groups, describe the direction of the effect.	N/A
	20c	Present results of all investigations of possible causes of heterogeneity among study results.	Results, pg6, table 1, pg12-15, narratives under each theme
	20d	Present results of all sensitivity analyses conducted to assess the robustness of the synthesized results.	Results, pg12-15, narratives under each theme
Reporting biases	21	Present assessments of risk of bias due to missing results (arising from reporting biases) for each synthesis assessed.	N/A
Certainty of evidence	22	Present assessments of certainty (or confidence) in the body of evidence for each outcome assessed.	N/A
**DISCUSSION**			

PRISMA 2020 Checklist

Section and Topic	Item #	Checklist item	Location where item is reported
Discussion	23a	Provide a general interpretation of the results in the context of other evidence.	Discussion, pg16-19
	23b	Discuss any limitations of the evidence included in the review.	Limitations of the study pg19
	23c	Discuss any limitations of the review processes used.	Limitations of the study pg19
	23d	Discuss implications of the results for practice, policy, and future research.	Limitations of the study pg19 prg4; Conclusions pg19
**OTHER INFORMATION**			
Registration and protocol	24a	Provide registration information for the review, including register name and registration number, or state that the review was not registered.	Method,pg4 prg4
	24b	Indicate where the review protocol can be accessed, or state that a protocol was not prepared.	Method,pg4 prg4
	24c	Describe and explain any amendments to information provided at registration or in the protocol.	N/A
Support	25	Describe sources of financial or non-financial support for the review, and the role of the funders or sponsors in the review.	Acknowledgement, pg20
Competing interests	26	Declare any competing interests of review authors.	Disclosure of Interest, pg20
Availability of data, code and other materials	27	Report which of the following are publicly available and where they can be found: template data collection forms; data extracted from included studies; data used for all analyses; analytic code; any other materials used in the review.	Data availability,pg20

Table S2. Inclusion and exclusion criteria of the selection of studies for the review

Characteristic	Inclusion criteria	Exclusion criteria
Population	NCD patients based on community	Patients in hospitals
Health services	1. Health services in community settings<br>2. Integrated, i.e., including an element of coordination among different levels and sites of care within and beyond the health sector	Hospital-based health services, services other than integrated health services
Outcomes	Effectiveness: prevalence, management, treatment and control of NCDs, quality of life<br>Acceptability: participation rate, satisfaction rate, compliance rate<br>Feasibility: coverage, staffing, time constraints<br>Efficiency: tertiary hospital visits (for common NCDs), expenditure (patients, insurance)	
Study design	Experimental (randomized controlled trials, quasi-randomized controlled trials, non-randomized clinical trials)<br>Quasi-experimental (interrupted time series, controlled before-after studies) and<br>Observational (cohort, case-control, cross-sectional, case series)<br>Qualitative studies	Reviews, meta-analyses, overviews, opinion pieces (i.e., commentaries, editorials, letters to editor)
Language	Articles published in English and Chinese	Articles published in other languages
Country	China	Any other country

Table S3. Electronic search strategy for databases

1) MEDLINE

I.	MEDLINE Via PubMed
1	"Delivery of Health Care, Integrated"[Mesh] OR “delivery of care” OR "delivery of healthcare" OR "Comprehensive Health Care"[Mesh] OR "comprehensive healthcare" OR "comprehensive care" OR "comprehensive health" OR "Continuity of Patient Care"[Mesh]OR "continuity of patient care" OR "continuity of care" OR "continuity of health" OR "continuity of healthcare" OR “cross sectoral care” OR "intersectoral care” OR “integrated care*”OR “integrated health” OR “integration of care” OR multi team OR “integrated service network” “multiagency care” OR multiteam OR “multi care” OR “integrated services” OR “intersectoral care” OR multicare OR “multi-agency care” OR “multi service” OR multiservice OR “multi program” OR multiprogram OR “multi programme” OR “multi delivery” OR multidelivery OR “multi management OR “outreach services”
2	“coordinated care” OR “co-ordinated care” OR coordinat* care OR "coordination of care" OR “care co-ordination” OR coordinat* services OR "coordination of services" OR coordinat* programmes OR coordinat* programs OR “coordination of programmes” OR “coordination of programs” OR “coordination of service delivery” OR coordinat* services OR “coordination of services” OR coordinat* service delivery OR coordinat* delivery OR “chains of care” OR “collaborative care” OR “care coordination” OR “care transition” OR “cooperative care”
3	“horizontal service delivery” OR “horizontal delivery” OR “horizontal care” OR “horizontal integration” OR “horizontal services” OR “horizontal management” OR “horizontal programmes” OR “horizontal programs” OR “vertical services” OR “vertical programmes” OR “vertical programs” OR “vertical care” OR “vertical service delivery” OR “vertical services” OR “vertical management” OR “vertical integration” OR “clinical integration “ OR “financial integration” OR “functional integration”
4	1 OR 2 OR 3
5	(community/).ti,ab,jw,nw.
6	Organization/
7	Society/
8	District/
9	5 OR 6 OR 7 OR 8
10	“Noncommunicable diseases” OR “Chronic diseases”
11	(hypertension or diabete* or cancer or COPD or cardiovascular disease*).ti,ab,jw,nw.
12	10 OR 11
13	“China”
14	4 AND 9 AND 12 AND 13 AND 14

2) EMBASE:

II.	EMBASE via Ovid
1	("Delivery of Health Care, Integrated"[Mesh] OR “delivery of care” OR "delivery of healthcare" OR "Comprehensive Health Care"[Mesh] OR "comprehensive healthcare" OR "comprehensive care" OR "comprehensive health" OR "Continuity of Patient Care"[Mesh]OR "continuity of patient care" OR "continuity of care" OR "continuity of health" OR "continuity of healthcare" OR “cross sectoral care” OR "intersectoral care” OR “integrated care*”OR “integrated health” OR “integration of care” OR multi team OR “integrated service network” “multiagency care” OR multiteam OR “multi care” OR “integrated services” OR “intersectoral care” OR multicare OR “multi-agency care” OR “multi service” OR multiservice OR “multi program” OR multiprogram OR “multi programme” OR “multi delivery” OR multidelivery OR “multi management OR “outreach services”).ti,ab,jx
2	(“coordinated care” OR “co-ordinated care” OR coordinat* care OR "coordination of care" OR “care co-ordination” OR coordinat* services OR "coordination of services" OR coordinat* programmes OR coordinat* programs OR “coordination of programmes” OR “coordination of programs” OR “coordination of service delivery” OR coordinat* services OR “coordination of services” OR coordinat* service delivery OR coordinat* delivery OR “chains of care” OR “collaborative care” OR “care coordination” OR “care transition” OR “cooperative care”).ti,ab,jx
3	(“horizontal service delivery” OR “horizontal delivery” OR “horizontal care” OR “horizontal integration” OR “horizontal services” OR “horizontal management” OR “horizontal programmes” OR “horizontal programs” OR “vertical services” OR “vertical programmes” OR “vertical programs” OR “vertical care” OR “vertical service delivery” OR “vertical services” OR “vertical management” OR “vertical integration” OR “clinical integration “ OR “financial integration” OR “functional integration”).ti,ab,jx
4	1 OR 2 OR 3
5	exp Community /
6	exp Organization /
7	exp Society /
8	exp District /
9	5 OR 6 OR 7 OR 8
10	“Noncommunicable diseases” OR “Chronic diseases”
11	(hypertension or diabete* or cancer or COPD or cardiovascular disease*).ti,ab,jw,nw.
12	10 OR 11
13	China
14	4 AND 9 AND 12 AND 13 AND 14

3) Chinese Index Medicus

III.	Chinese Index Medicus
1	Title or keyword = (community or demonstration district or community care or community health or community services)
2	Title or keyword = (chronic disease or diabetes or hypertension or heart disease or chronic obstructive pulmonary disease or stroke or cardiovascular disease)
3	Title or keyword = (integration or service or management or policy or model)
4	China
5	1 and 2 and 3 and 4 and 5
